# Different types of facial description alter the confidence–accuracy relationship

**DOI:** 10.1038/s41598-026-49407-0

**Published:** 2026-05-05

**Authors:** Dawn R. Weatherford, Curt A. Carlson, Lacy E. Krueger, Christopher R. Williams, Roman M. Pardo, Maria A. Carlson

**Affiliations:** 1 Department of Health and Behavioral Sciences, Texas A&M University-San Antonio, 1 University Way, San Antonio, TX 78224 USA; 2 East Texas A&M University, Commerce, TX USA; 3 Arkansas State University, Jonesboro, AR USA

**Keywords:** Verbal facilitation, Facial recognition, Facial description, Confidence-accuracy relationship, Task demands, Holistic processing, Human behaviour, Long-term memory

## Abstract

Eyewitnesses commonly provide facial descriptions after the commission of a crime. Although research suggests that this description may improve memory (i.e., verbal facilitation), less is known about what types of descriptions (e.g., parts-based featural, trait-based holistic) are most suitable to maximize accuracy. To address this gap, participants studied two blocks of 12 faces (i.e., 24 total study faces) and performed either a Description (i.e., featural, trait) or Control (i.e., counting, addition) task after each (condition names representing block order). Following a short distractor, participants completed two types of recognition memory tests. The inclusion test instructed participants to respond “yes” to all studied faces in both blocks and “no” to new faces. Conversely, the exclusion test instructed participants to respond “yes” to studied faces in Block 1 and “no” to studied faces in Block 2 and new faces. Participants subsequently provided confidence and source-memory judgments. With respect to accuracy, either type of Description (as opposed to Control) facilitated memory. Participants who completed a Description-Control or Control-Description block order combination outperformed Control-Control participants. Participants’ exclusion test performance showed a similar description advantage; Description-Control outperformed Control-Description and Control-Control. Although a general description advantage emerged for overall accuracy, the confidence-accuracy relationship manifested in different ways. The number of trait descriptors increased accuracy without affecting confidence. The number of featural descriptors increased confidence without affecting accuracy. We discuss these findings and their implications for guiding person descriptions for different types of eyewitnesses with and without advanced preparation of witnessing a criminal event.

**Supplementary Information:**

The online version contains supplementary material available at 10.1038/s41598-026-49407-0.

## Introduction

Facial recognition is important across a variety of social and forensic situations. Individuals possess a robust ability to identify familiar faces^1^ despite within-person variability attributable to natural changes over time (e.g., aging, weight, skin tone; e.g.^2,3^) and surface-level differences (e.g., cosmetics, hairstyle, jewelry and accessories). Observers can reliably extract identity-based information even under poor encoding conditions (e.g., lighting^4^, distance^5^, time^6^). This superior skill, however, does not wholly extend to unfamiliar faces^7^. Remembering sufficient information to identify an unknown individual is challenging and error prone^8^. Unfamiliar face processing does not benefit from the large body of semantic and visual information stored in memory for friends, family, and celebrities (i.e., Person Identity Nodes^9^).

Nevertheless, individuals routinely need to remember and identify unknown persons. Within a forensic context, eyewitnesses often view crimes perpetrated by strangers. Subsequently, law enforcement solicits information such a verbal description (e.g., cognitive interview^10^ in order to identify a suspect and create a lineup. The relationship between verbal descriptions and lineup identification, however, is delicate. Research demonstrates that although providing person descriptions sometimes facilitates face memory and recognition (i.e., verbal facilitation^11^), it may also hinder face memory and recognition (i.e., verbal overshadowing; VOE^10^. Regarding the latter, Meissner and Brigham’s^12^ meta-analysis of verbal overshadowing literature suggested that, when observed, these effect sizes are small and more likely to occur when there is no delay (or a minimal delay) between verbalization and identification. In other words, when memory for the verbal representation of the face overshadows memory for the visual representation because the description is recently activated in working memory, that description may impair identification performance. With longer delays between verbalization and identification tasks, however, verbal facilitation was more likely to occur. Moreover, researchers also note that verbal overshadowing is more pronounced with delays between studying the visual representation of the face and subsequently generating a verbal representation. Namely, descriptions immediately following stimuli presentation showed less verbal overshadowing (and greater likelihood of facilitation) than if a delay occurred between viewing and description. In sum, less intervening time between facial study and description and more intervening time between facial description and identification were more likely to produce facilitation.

Given that verbalizations can facilitate identification under these circumstances, it is crucial to understand *which type(s)* of descriptions promote facilitation (e.g.,^13,14^). Strategic interviewing techniques and advanced training may elicit the types of person descriptions that would produce facilitation. Although eyewitnesses typically provide short and impoverished person descriptions^13^, techniques akin to the cognitive interview may encourage eyewitnesses to expand upon their descriptions so as to increase their likelihood of successful identification. Likewise, individuals who may more routinely view perpetrators (e.g., security and surveillance personnel, bank tellers, police officers) may benefit from professional education to practice and apply particular types of person descriptions as a strategy. To address this gap in the literature and promote evidence-based strategies that are practicable in real-world settings, this paper first explores the empirical evidence and theoretical accounts of verbal facilitation before transitioning to the current study designed to uncover additional potential with verbal reports.

### Why does facial description facilitate memory?

The typical verbal facilitation (VF) paradigm directs participants to study a series of faces and describe each face (or not) directly after its presentation. Following a short distractor period, participants are better able to identify faces followed by a description task than faces followed by a control task (e.g., counting backwards^11,15,16^), no task (e.g., viewing a blank screen for an equivalent amount of time^17^) or another face-related non-description task (e.g., providing pleasantness ratings, visualizing^17^); reading facial descriptions generated by others^18^. Critically, verbal facilitation occurs even when participants are unaware that they will need to describe a face until after it is removed from view^17^, akin to an eyewitness who does not know that a crime is about to occur.

Given its theoretical and practical significance, researchers have sought to identify the source of this VF effect (for a meta-analysis, see^14^). Two principal possibilities have emerged: (1) *description enhances encoding processes recruited during study* such as more distributed visual scanning^18–20^; deeper semantic elaboration^21,22^ and enhanced face-specific benefits (e.g., similarly attributable to configural, holistic, and global processing^21,22^); (2) *description enriches the stored memory representation with retrieval “tags”* supporting subsequent recognition memory^18,22^.

These two sources of VF are not mutually exclusive. Applied to an eyewitness memory context, the first possibility suggests that description supports enhanced encoding of information about the perpetrator and the criminal event. The second possibility suggests that the description might only increase correct recognition when system variables adequately trigger retrieval of those improved memories. In other words, having a strong memory alone will not necessarily benefit recognition if the testing conditions do not encourage its use.

Weatherford et al.^11^ also argued that two different theoretical explanations are unnecessary. They merged the relevance of both encoding and retrieval factors by proposing a more parsimonious *expanded recollection* account. By their account, description enhances recollection of a wide variety of episodic information (e.g., perceptual, semantic, and descriptive). To produce facilitation, however, recollecting that information must be useful in making a recognition decision. In such instances, recollection involves the conscious re-experiencing of elements of the study experience to support both the correct identification of described individuals and the correct rejection of similar-looking individuals who might evoke a false sense of familiarity^23,24^.

In the work that supported this position, participants described or counted backwards after each face in a series^11^. As in Jones et al.^16,17^, participants were unaware about which task would be prompted until after the face was removed. After the study phase, memory was tested with studied, new, and conjunction faces. Conjunction faces recombined elements of two studied faces (i.e., the eyes and mouth studied in one intact face were recombined with the nose and facial shape studied in another intact face). Participants were made aware that these faces would be included in the test and received two different sets of instructions about how to respond to them. Under inclusion instructions, participants were directed to include conjunction faces by responding “yes” to any studied or conjunction faces, while responding “no” to any new faces. Under exclusion instructions, participants were directed to exclude conjunction faces by responding “yes” only to studied faces, while responding “no” to any conjunction or new faces.

In Experiment 1, participants performed two different types of control tasks (i.e., counting and addition). Not only did neither task facilitate recognition memory, but participants were unable to flexibly respond to the conjunction faces. Exclusion and Inclusion test patterns were strikingly similar (see also^25^). In Experiment 2, however, description (but not counting) increased the proportion of “yes” responses to studied faces relative to new faces (i.e., intact-new discrimination) – replicating the classic verbal facilitation effect. Notably, *description enhanced recollection*. Conjunction faces that recombined elements of two described study faces were more likely to be included in the “yes” responses under the inclusion test instructions (as evidenced by conjunction-new discrimination) and excluded from the “yes” responses under exclusion test instructions (as evidenced by intact-conjunction discrimination).

Although VF is a robust finding across a variety of different paradigms, ambiguity in the source of this effect has obscured straightforward translation to an eyewitness context. Simply knowing that verbal reports can strengthen memories does not inform practice, especially if the type of reports that eyewitnesses typically provide lack sufficient detail (e.g.,^13^). Instead, investigations should explore not only when to solicit reports from eyewitnesses (e.g.,^12^), but also about what types of information to include in those reports. As is evidenced by the proliferation of data-driven theoretical accounts above, the role of description quantity and quality is not clear.

### What types of facial description facilitate memory?

Many researchers have defined the total number of descriptors as a measure of quantity and description type (e.g., featural, trait, configural) as a measure of quality. Meta-analytic evidence from^14^ and more recent investigations remain mixed. Some evidence suggests a positive relationship between recognition accuracy and total number of descriptors (which tend to increase with description duration), whereas other evidence does not. Relatedly, some evidence suggests that holistic/configural/global descriptors (e.g., perceptually-derived judgments of honesty, attractiveness, and personality) that reinforce face-specific cognitive mechanisms improve recognition accuracy. Other findings suggest no differences from featural/parts-based descriptions (e.g., eyes, nose, mouth). How can we reconcile these seemingly disparate findings?

Appealing again to the *expanded recollection* account, descriptions strengthen memories that affect retrieval *only when testing conditions require their use*. In this respect, the expanded recollection account does not make universal predictions about the role of different types of verbal descriptors in systematically altering encoding and retrieval processes. Instead, this account acknowledges that generating any type of description will support more robust encoding that enhances recollection of many different aspects of the face-viewing experience. Subsequently, it is important to consider two aspects of eyewitness memory encoding and retrieval experience as nearly universally true.

#### Eyewitnesses’ featural descriptions are necessary to construct lineups

First, eyewitnesses must report featural information. Objective descriptions of facial elements (e.g., shape, size, color, configuration) are typically necessary to identify a suspect (e.g.,^13^). Although subjective descriptions that maintain the holistic representation and support automatic face processing may preserve more intact face memory (e.g.,^26^), police officers would have little use for eyewitnesses’ impressions of attractiveness. Promisingly, not all research suggests a deleterious effect of featural description. On the contrary, many theoretical accounts outside of the description-identification literature suggest an important role for featural information in suspect identification. The dual-route hypothesis^27^, for instance, proposes that face processing follows two routes: featural and holistic. Whereas the featural route is more bottom up and deliberate, it is an equally viable way to remember faces as the holistic route that is more top-down and automatic.

Further, multiple-face lineups, such as simultaneous or sequential, often include description-matched lures. In other words, an eyewitness’s featural description has limited use if it applies to each possible face in the lineup. Wixted and Mickes^28^ argue that the inclusion of description-matched lures promotes better lineup decisions through a process called Diagnostic Feature Detection (DFD; see also^29^. If all members of a fair lineup possess each of those description-matched features, DFD predicts that eyewitnesses can discount features that are replicated across every face in the lineup. Instead, correct decisions will rely upon potentially non-overlapping visual information that pops out as unique in the array, subsequently triggering memory for the target face (even if the diagnostic feature was not articulated in the verbal report).

To align with these real-world circumstances, multiple-face paradigms must establish verbalizations’ effects when controlling for facial similarity. If description only benefits memory for faces that are dissimilar (e.g., different ages, genders, and races) or distinctive (e.g., tattoos^30,31^; abnormally sized features^32^; accessories^1,33^, then that paradigm fails to translate to eyewitness memory. As such, the current experiment used a multiple-face study and test sequence comprised of grayscale images of non-distinctive, young, white males who may all reasonably be placed in the same lineup as one another (see Materials for more information). If verbalization benefits memory above and beyond the description itself, then our paradigm should still be able to detect those differences despite highly similar faces.

#### Eyewitness confidence may also be informative

Another consideration is the role of confidence. Law enforcement officers often rely upon eyewitness testimony to corroborate other physical evidence. Although the preponderance of evidence may make a strong case for a conviction, irrefutable evidence about culprit guilt is often unknown. In this respect, eyewitness confidence can sometimes serve as a proxy for accuracy. Police officers, attorneys, and jurors may be easily swayed by a confident eyewitness (e.g.,^34,35^). Nevertheless, manipulations that inflate confidence without affecting accuracy and vice-versa are well-documented (see the Innocence Project for real-world examples). Any VF study that seeks to translate theory to practice, therefore, needs to account for and identify factors that either enhance or disrupt the confidence-accuracy (C-A) relationship for faces (e.g.,^36^).

As with overall accuracy, the VF literature is less clear in its predictions of the effect of description quantity and quality on the C-A relationship. Outside of the verbalization literature, Reinitz et al.^37^ proposed an account that may unify our interpretation of these documented C-A effects. In their multidimensional model, the authors articulated the routes by which familiarity-based and feature-based encoding factors affect cognitive, metacognitive, and decision-making mechanisms. On one hand, familiarity-based encoding manipulations (e.g., duration, repetition) improve memory strength (*cognitive*) without affecting certainty (*metacognitive*). Therefore, recognition and confidence judgments may be well-calibrated such that increased memory strength has similar effects on both types of decisions. On the other hand, feature-based encoding manipulations affect memory strength (*cognitive*) and certainty (*metacognitive*). Unlike memory strength, certainty singularly affects confidence judgments without affecting recognition decisions. Feature-based manipulations, therefore, disrupt the C-A relationship by inflating confidence.

Mapping these broader theoretical predictions to the verbalization paradigm is clear-cut. Trait descriptors, argued to rely upon a familiarity-based holistic process, should increase both accuracy and confidence in similar ways. Featural descriptors, on the other hand, should inflate confidence more so than accuracy.

## The present study

To study the sources and outcomes of VF in ways that will test predictions of the *expanded recollection*^11^ and *multidimensional*^18^ accounts, we systematically manipulated the study and test phases of a multiple-face recognition paradigm. In the study phase, participants completed a different task in each of two study blocks. Participants were assigned to one between-participants factor of Block Order (Control-Control, Control-Description, or Description-Control). The *Control-Control* participants completed Counting Backwards and Addition, counterbalanced across participants. The *Control-Description* participants completed a Counting Backwards task in the first block and either a Featural Description or Trait Description task in the second block, counterbalanced across participants. Finally, *Description-Control* participants completed a Featural Description or Trait Description task in the first block, counterbalanced across participants, and a Counting Backwards task in the second block.

In the test phrase, all participants completed two different types of recognition tests: Inclusion and Exclusion. During the inclusion test, participants were instructed to respond “yes” to all faces studied from Block 1 and Block 2. During the exclusion test, participants were instructed to respond “yes” only to faces studied in Block 1. Both types of tests also included source memory prompts, such that participants were asked to identify the block of each presented face (i.e., Block 1, Block 2, or new).

These study and test manipulations allowed us to calculate recognition accuracy, confidence-accuracy calibration, and source memory for faces that either were or were not described. First, in line with the *expanded recollection* account^11^, we expected that both the *Description-Control* and *Control-Description* groups would have higher recognition accuracy than the *Control-Control* group on the Inclusion test. However, we expected that the *Description-Control* group would have higher recognition accuracy than the *Control-Description* and *Control-Control* groups on the Exclusion test.

Second, in line with the *multidimensional* account^18^, we anticipated that trait description would improve the confidence-accuracy relationship relative to featural description, as trait description should contribute to accuracy without inflating confidence. As described in detail below, we conducted calibration analysis (e.g.,^38^) and we portray calibration curves to test this prediction. Specifically, we expected that trait description would yield a stronger confidence-accuracy relationship in two ways: (a) superior calibration (i.e., *C* statistic closer to zero) and (b) confidence would be a stronger postdictor of accuracy as shown with the *z*-statistic comparing proportion correct between the lowest and highest confidence bin^39^.

We also anticipated that different types of description would affect source memory in line with Reinitz et al.’s^37,40^ multidimensional model. Specifically, featural description should strengthen the featural route and trait description should strengthen the familiarity route to face recognition in ways that are not revealed by aggregate comparisons of recognition accuracy or confidence-accuracy. As such, we compared how the number and type of person descriptions affected our dependent variables on a trial-by-trial basis. We predicted that the number of featural descriptors would positively correlate with confidence, but not with accuracy (i.e., as measured by source prompts for Block 1, Block 2, or new). Likewise, the number of trait descriptors would positively correlate with accuracy, but not confidence.

## Methods

### Participants

Native English-speaking undergraduate students (*N* = 209; *M*_age_ = 24.51, *SD*_age_ = 8.83; 154 women and 55 men) with normal or corrected-to-normal vision participated in exchange for partial course credit. Self- reported race reflected a diverse sample (7 Asian/Asian-American, 59 Black/African American, 20 Hispanic/Latino, 115 White/Caucasian, 5 other, and 3 chose not to answer). All procedures were approved by the Institutional Review Board of East Texas A&M University. Further, all methods were performed in accordance with the Code of Federal Regulations, Title 45 Part 4: Protection of Human Subjects (45 CFR Part 46).

### Materials

**Fig. 1:**
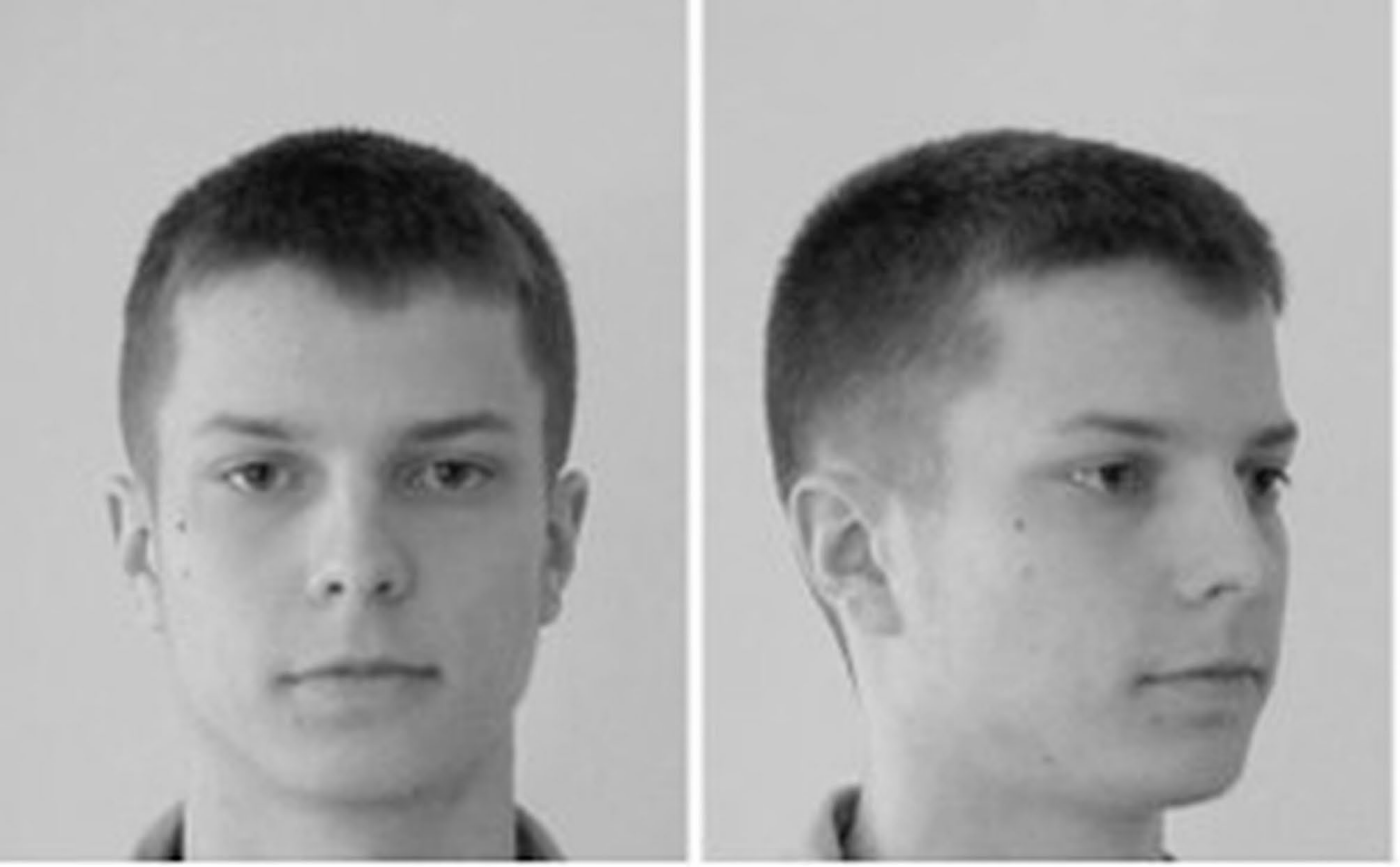
Example Experimental Stimuli. *Note*: Example representation of faces presented in frontal view at study (left) and three-quarters view at test (right).

To ensure that data reflect face processing and not image processing^41^, we included two different orientations (frontal view at study and three-quarters view at test) of Caucasian males (Computer Vision Laboratory, Faculty of Computer and Information Sciences, University of Ljubljana, Slovenia, http://lrv.fri.uni-lj.si/facedb.html; see Fig. 1 that sample identities for which written permission to publish images was secured by the CVL). Only photographs without large jewelry, facial hair, and glasses were included. Adobe Photoshop CS2 (www.adobe.com) was used to crop, convert to grayscale, and edit out small jewelry (e.g., earring, necklace) that could be used as featural cues.

### Procedure

#### Study phase

After providing informed consent, participants completed the experiment using E-prime version 2.0^42^. Participants were told that they would view a series of faces, one at a time, and be asked to perform a task after each face was removed from the screen. The study phase included two different blocks of study faces, such that each block contained 14 faces (i.e., 28 study faces in all, which included 2 buffer faces 1 in the beginning and 1 at the end to avoid primacy or recency effects). After each face was removed from the screen, participants were given 30 s to perform the encoding task. Encoding tasks varied by block, such that participants performed either a control or description task.

To ensure adherence to encoding task instructions, participants performed three practice trials with corrective feedback prior to each block. The *counting* control task instructions encouraged participants to count back from a random three-digit number in intervals of three while the number appeared on the screen. Then, participants saw a prompt to type the number to which they counted. The *addition* control task instructions encouraged participants to solve simple addition problems on a piece of paper. After 28 s, participants saw a black screen and heard a ringing tone, which reminded them to stop their calculations and redirect their attention to the computer monitor in preparation for the next face. The *featural* description task instructions encouraged participants to “describe each face in terms of its features such as eyes, nose, and mouth.” The *trait* description task instructions encouraged participants to “describe each face in terms of the person’s likely personality type, honesty, and attractiveness.” Both sets of description instructions encouraged participants to “be as descriptive as possible and continue typing your description until the next screen appears.” After each encoding task, participants viewed a white screen for 2.5 s before seeing the next face.

#### Distractor phase

After the completion of the study phase, participants performed a distractor task (Sudoku) for 5 min.

#### Recognition memory and source monitoring phase

Participants then performed two self-paced recognition tests, each with a different set of instructions (inclusion or exclusion; order counterbalanced across participants). For each test, participants were presented with six faces from Block 1, six faces from Block 2, and 12 new faces. In the inclusion test, participants were instructed to respond “yes” to any face that appeared in either of the two blocks and “no” to all other faces. For the exclusion test, participants were instructed to reply “yes” to faces only if they appeared in Block 1 and “no” to all other faces. Participants made recognition decisions by pressing either the “y” or “n” key. Participants were then asked to provide a confidence rating on a scale of 1 (“not at all confident”) to 6 (“very confident”).

After the confidence rating, participants were probed to identify the source of their memory for certain faces. During the inclusion test, faces to which participants responded “y” were followed by a prompt to press “1” if they thought they studied the face during Block 1 or “2” if they thought they studied the face during Block 2. No additional information was requested if participants responded with “n.” During the exclusion test, faces to which participants responded “n” were followed by a prompt to press “2” if they thought they studied the face during Block 2 or “0” if they thought they did not study the face. No additional information was requested if participants responded with “y.”

## Results

To reduce error variance due to low motivation or noncompliance with test instructions, we excluded participants who performed at or below chance i.e., proportions of “yes” responses to new faces greater than proportion of “yes” responses to old faces^15^. This performance cutoff resulted in the exclusion of 31 participants.

To address our VF predictions, we considered differences in recognition accuracy (i.e., signal detection indices calculated from yes/no recognition responses), the confidence-accuracy relationship (i.e., calibration and *z*-scores based on proportion correct calculated from the 1 to 6 confidence bins), and the differential effects of featural and trait description on source memory (i.e., trial-by-trial correlations between number of descriptors and both 1–6 confidence responses and block source responses).

### Recognition accuracy

**Fig. 2:**
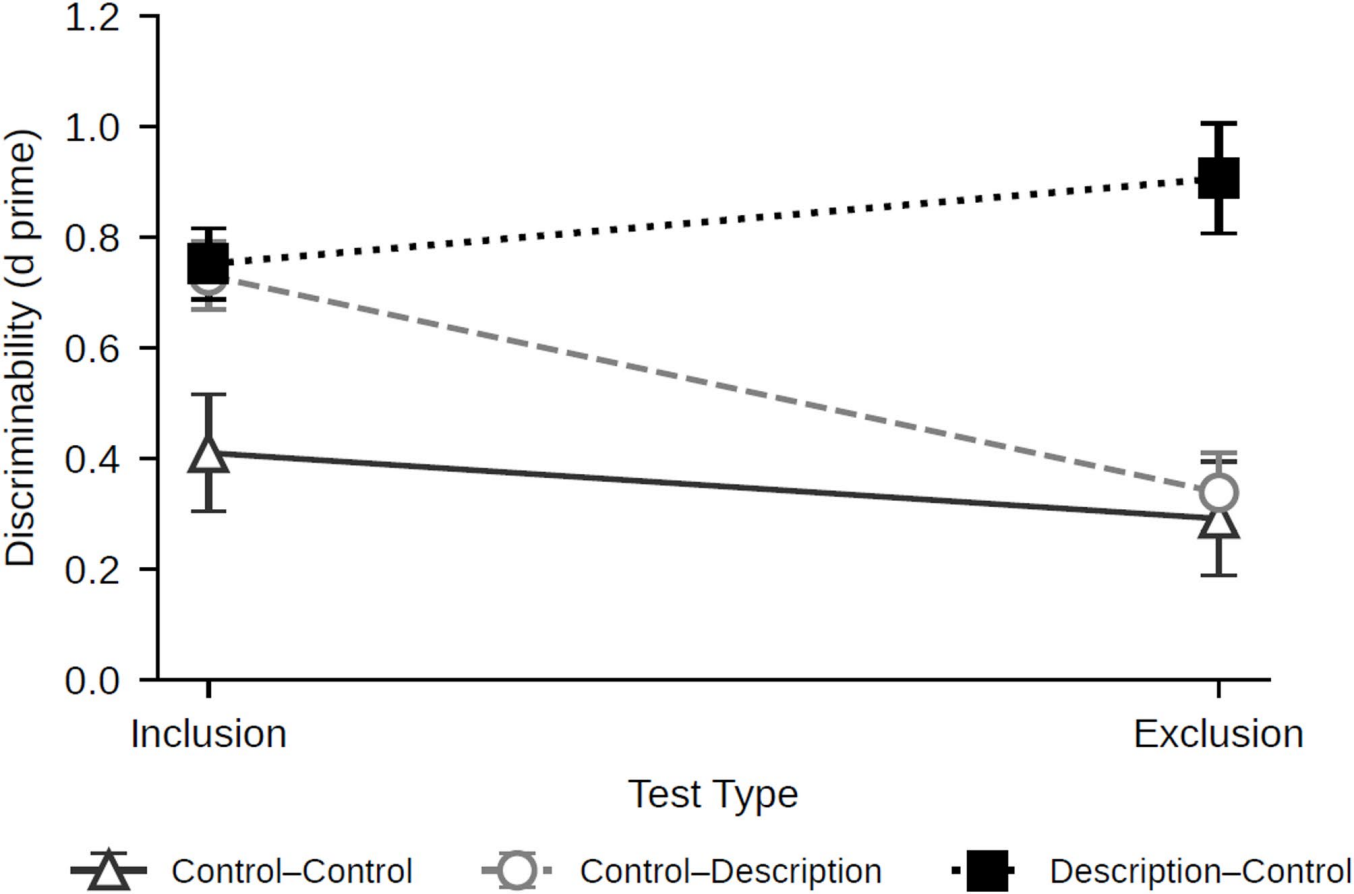
Discriminability by Test Type and Description Condition.* Note*. Error bars represent 95% confidence intervals.

Results are presented in Fig. 2. A 3 (Block Order – between: Control-Control, Control-Description, or Description-Control) x 2 (Test Type – within: Inclusion and Exclusion) mixed methods factorial ANOVA on *d’* revealed a main effect of Block Order, *F*(2, 175) = 17.54, *p* < .001, *η*^*2*^_*p*_ = 0.167), and no main effect of Test Type *F*(1, 175) = 2.30, *p = ns*, *η*^*2*^_*p*_ = 0.013). Importantly, there was an interaction between Block Order and Test Type, *F*(2, 175) = 5.76, *p* = .004, *η*^*2*^_*p*_ = 0.062. Post hoc tests using Bonferroni correction (all *p*s < 0.001) confirmed the theoretical predictions of the *expanded recollection* account that Description-Control and Control-Description would improve accuracy over Control-Control for inclusion, yet only Description-Control would increase accuracy over Control-Control for exclusion.

For the inclusion test, the Control-Control condition (*M* = 0.41, *SEM* = 0.10) was significantly lower than both the Control-Description (*M* = 0.73, *SEM* = 0.07) and Description-Control (*M* = 0.75, *SEM* = 0.06) conditions, which did not differ from each other. These data suggest that, as description facilitates memory for faces, participants who described any study faces could use their superior memory performance to respond “yes” to faces as directed.

For the exclusion test, however, participants are tasked with only responding “yes” to faces from the first block. In line with our predictions, Description-Control participants (*M* = 0.91, *SEM* = 0.08) outperformed both Control-Control (*M* = 0.29, *SEM* = 0.13) and Control-Description (*M* = 0.34, *SEM* = 0.09), which did not differ from each other. These data suggest that, as description facilitates memories for faces described in the first block, Description-Control participants were more readily able to follow the exclusion test instructions. Complete statistical analyses, including additional source memory and confidence metrics are available in the supplementary materials. These additional analyses extend, but do not alter, our main findings.

### Confidence–accuracy relationship

**Fig. 3:**
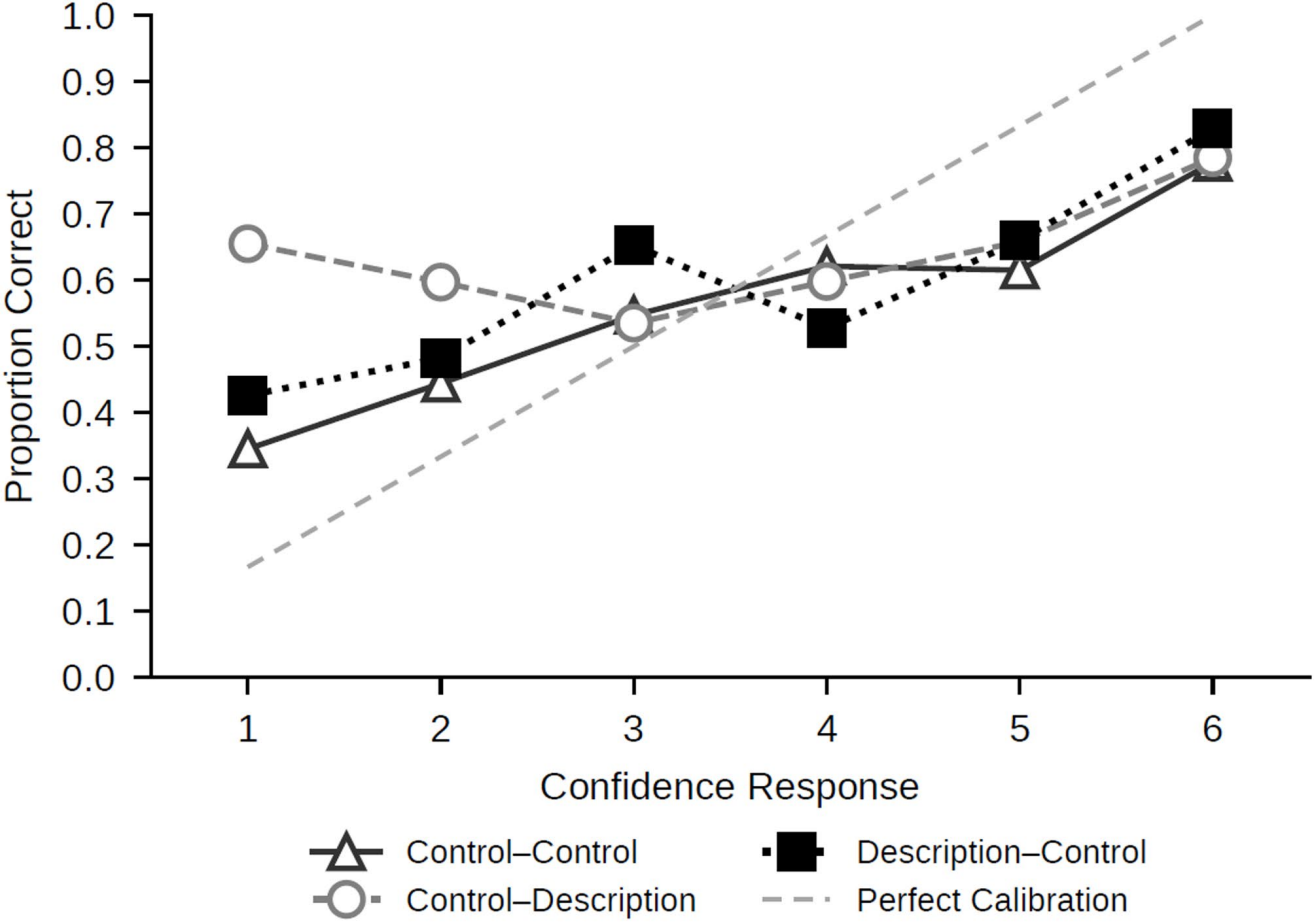
Inclusion Test: Proportion Correct by Confidence Response and Condition.

**Fig. 4:**
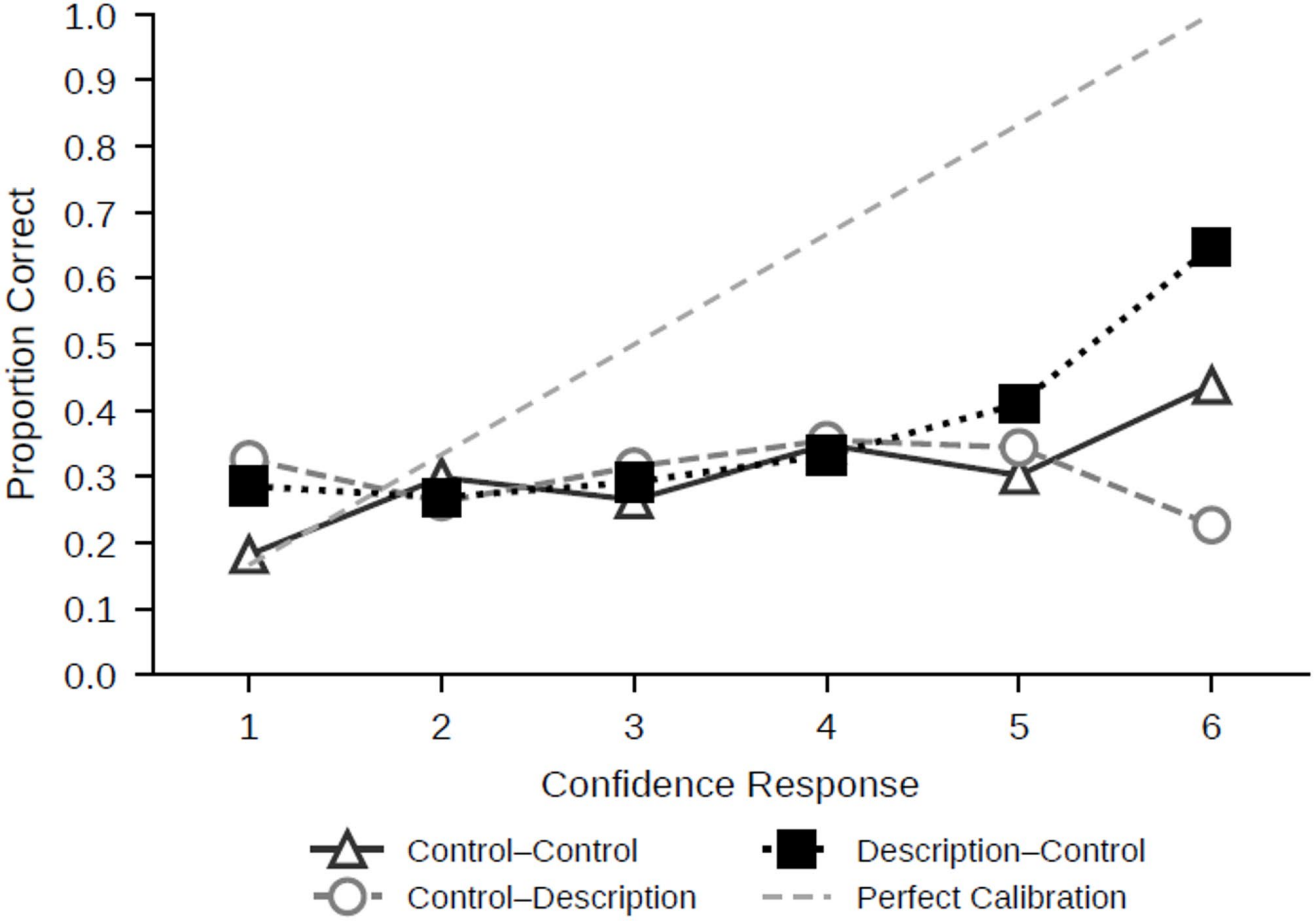
Exclusion Test: Proportion Correct by Confidence Response and Condition.

Our second prediction was that trait description conditions would produce a stronger C-A relationship compared to featural description or control conditions. This relationship is often depicted with calibration curves (e.g.^38,43^), which we present below in Figs. 3 and 4. There are two aspects to note from each graph: (a) calibration, or how closely a curve aligns with the dashed line indicating perfect calibration (ranges from 0 to 1, with values closer to 0 representing better calibration; see Table 1); and (b) the increase in proportion correct from the lowest to highest confidence bin. Participants in a given condition are well-calibrated if their confidence tends to align with their accuracy (e.g., confidence of 1 = about 17% accurate, 2 = about 34% accurate, and so forth), and confidence is a better postdictor of accuracy as the proportion correct in the highest confidence bin more greatly exceeds the proportion correct in the lowest confidence bin (evaluated statistically with a *z*-test of proportions, see Table 1).

**Table 1:** Calibration and z-scores per condition.

Condition	C (95% CI)	*z* (*p*)
Control-control inclusion	0.0159 (0.0157, 0.0161)	3.74 (< 0.001)
Control-featural inclusion	0.0200 (0.0194, 0.0206)	1.52 (0.13)
Control-trait inclusion	0.0436 (0.0430, 0.0442)	0.65 (0.52)
Featural-control inclusion	0.0219 (0.0213, 0.0225)	5.43 (< 0.001)
Trait-control inclusion	0.0244 (0.0238, 0.0250)	4.03 (< 0.001)
Control-control exclusion	0.0928 (0.0922, 0.0934)	1.99 (0.047)
Control-featural exclusion	0.1151 (0.1141, 0.1161)	0.29 (0.77)
Control-trait exclusion	0.1194 (0.1184, 0.1174)	0.81 (0.42)
Featural-control exclusion	0.1070 (0.1060, 0.1080)	1.78 (0.075)
Trait-control exclusion	0.0665 (0.0655, 0.0675)	3.73 (< 0.001)

Note: Inferential Confidence Intervals in parentheses, based on modified jackknife procedure from Mosteller and Tukey (1968). C = calibration, with numbers closer to zero representing better calibration; z score represents the statistical difference between the proportion correct at the lowest versus highest confidence bin, as an indication of the value of confidence in postdicting accuracy.

The first aspect of Figs. 3 and 4 to focus on is worse performance for Exclusion (Fig. 4) compared to Inclusion (Fig. 3). All Exclusion curves are lower on the y-axis, which is a simple outcome of the more difficult task. Specifically, there are many more false alarms possible for Exclusion (new faces + Block 2 faces) compared to Inclusion (only new faces), which naturally drives down proportion correct (hits/hits+false alarms). This greater task difficulty could have contributed to the worse C-A relationship overall for Exclusion compared to Inclusion. As shown in Table 1, Inclusion is superior in terms of both calibration (as values are closer to zero) and *z*, with higher values representing a greater increase in accuracy from lowest to highest confidence bin.

In terms of our hypotheses, we expected an advantage for trait over featural description or control. This pattern was not supported with Inclusion but it was for Exclusion, as the Trait-Control condition yielded the best calibration and *z*-score. Critically, for the Exclusion condition participants need to indicate “yes” for only Block 1 faces, and in this condition those are the faces that were described based on traits. As shown in Fig. 3, this condition clearly has the best performance at the highest level of confidence. There is no comparable separation of conditions for Inclusion. In other words, in support of the *multidimensional* account, when testing conditions (i.e., exclusion instructions) required use of the verbal facilitation from describing the faces in terms of traits, there was the strongest C-A relationship, compared to other Exclusion conditions.

### Source accuracy and confidence by descriptor type

After having established overall patterns regarding the effect of description on memory for faces, we investigated a more fine-grained relationship between memory for each face and its corresponding description. Regression analyses allowed us to use the source recognition responses to interrogate the claims of the Reinitz et al.’s^37,40^ *multidimensional model* in ways that binary yes/no judgements may not. That is to say, description-level analysis may be more directly comparable to eyewitness descriptions (as discussed in greater detail in the General Discussion).

As is typical in the facial description literature (for a review, see^14^, we coded the quality and quantity of typed descriptions. Two independent, naïve judges tabulated the number of featural, trait, and configural descriptors for each description. These values were then summed, less any overlapping descriptors (e.g., “Almond shaped eyes set far apart from each other” would represent both a featural and configural descriptor), to generate a total number of descriptors per face. To assure a high degree of inter-rater reliability, both judges coded all verbalizations for the same two faces across both encoding tasks with α > .90 on all descriptor categories. The remaining faces were then rated independently by both raters. In line with Brown and Lloyd-Jones^22,44^, featural descriptions consisted of verbalizations about isolated facial features such as size and shape of eyes, nose, mouth, eyebrows, forehead, hair, and chin. Trait descriptors consisted of verbalizations about personality (e.g., “A trouble maker,” “happy go lucky,” “he deals with whatever hand of cards life deals him”), honesty (e.g., “he’s not up to anything good,” “does everything by the book”), attractiveness (e.g., “lacking in looks,” “very handsome”), and other social judgments (e.g., “runs a pizza shop or GameStop,” “plays sports”). Configural descriptors noted spatial distance between features in a face (e.g., “large space between bottom of nose and top of mouth”). This last category of configural descriptors did not predict any outcome variables and we do not consider it further beyond Table 2, which reports descriptive information about the frequency and types of information aggregated across valid trials.

**Table 2:** Descriptive statistics for descriptor type by description condition.

Descriptor		Featural	Configural	Trait	Total
	*N*	*M(SD)*	*M(SD)*	*M(SD)*	*M(SD)*
Condition					
Featural	850	1.76(1.91)	0.74(0.82)	0.04(0.20)	4.29(1.5)
Trait	814	0.09(0.35)	0.08(0.28)	2.82(1.3)	3.5(1.2)

We conducted linear regression analyses to evaluate whether the quantity of featural and trait descriptors differentially affected confidence and source accuracy^37,40^ (see Table 3). There was a positive relationship between confidence and featural descriptors (*r* = .12, adjusted *R*^*2*^ *=* 0.10, *F*(1, 849) = 12.27, *MS*_*e*_ = 2.11, *p* < .001), but not trait descriptors (*F*(1, 813) = 0.21, *MS*_*e*_ = 2.29). There was also a positive relationship between trait descriptors and source accuracy (as measured by follow up source judgments of “1” for Block 1, “2” for Block 2, and “0” for New) for both the inclusion (*r* = .66, adjusted *R*^*2*^ *=* 0.44, *F*(1, 98) = 77.25, *MS*_*e*_ = 0.05, *p* < .001) and exclusion tests (*r* = .64, adjusted *R*^*2*^ *=* 0.40, *F*(1, 98) = 65.73, *MS*_*e*_ = 0.07, *p* < .001). There was no relationship between featural descriptors and source accuracy on the inclusion (*F*(1, 69) = 0.23, *MS*_*e*_ = 0.06) or exclusion tests (*F*(1, 69) = 2.66, *MS*_*e*_ = 0.08).

**Table 3:** Regression coefficients of descriptor type and test on source accuracy and confidence.

Variable	Source accuracy					Confidence				
	*t*	B	β	SE	*p*	*t*	B	β	SE	*p*
Featural						3.50*	0.09	0.12	0.03	< 0.001
Inclusion	0.48	0.00	0.06	0.01	0.63					
Exclusion	1.63	0.00	0.19	0.01	0.11					
Trait						0.46	0.02	0.02	0.03	0.65
Inclusion	8.79*	0.02	0.66	0.01	< 0.001					
Exclusion	8.11*	0.02	0.63	0.01	< 0.001					

## General discussion

We established that description facilitated recognition memory for faces. Our findings support the *expanded recollection account* to extend the work of Weatherford et al.^11^. In both our work and theirs, featural and trait descriptions produced similarly superior performance on a recognition test compared to a control task. Given appropriate task demands (such as inclusion and exclusion test instructions in this case), description allowed participants to use their strengthened memories to recognize studied faces while rejecting new faces.

Additionally, our paradigm went further by incorporating a block source-monitoring task that followed the recognition decision and confidence judgement. This additional set of prompts allowed us to test the correspondence between individual descriptions and memory for each face. Despite equivalent discrimination performance between featural and trait description on yes/no recognition accuracy, verbal quality versus quantity differentially predicted source accuracy and confidence: a) trait description produced a stronger C-A relationship under Exclusion test instructions than featural or control, and b) additional featural descriptors increased confidence, but not source accuracy; additional trait descriptors increased source accuracy, but not confidence. These findings nicely align with the *multidimensional* account proposed by Reinitz et al.^37,40^.

Having supported the theoretical predictions of the *expanded recollection* and *multidimensional* accounts, how might these theories inform best practices for eyewitnesses and the law enforcement officers who shepherd them through the criminological process?

### Verbalization as a system variable to enhance eyewitness memory

Eyewitnesses customarily provide descriptions to aid law enforcement in locating a suspect and creating an appropriate lineup. In some instances, eyewitnesses have no reasonable expectation that they will soon view a crime. In such circumstances, law enforcement may play a key role in soliciting these descriptions to overcome the limited value of vague, impoverished descriptions that eyewitnesses typically produce (e.g.,^45^). In other instances, security professionals may have a more reasonable expectation that they could become eyewitnesses. In this set of circumstances, it may be more practicable to train such individuals to capitalize on strategies that instantiate verbal facilitation and avoid verbal overshadowing^11,44^.

If the contents of either type of eyewitnesses’ description affect confidence and accuracy, then additional research using both basic and applied paradigms should investigate the complex interplay suggested by Reinitz et al.’s^37,40^ *multidimensional model*. Our results suggest that eyewitnesses may benefit from including both featural and trait descriptors in their person descriptions. Although featural descriptors may be more helpful in apprehending a suspect (as features are visually bound and more objective, consensus-driven perceptual attributes of a face), lengthening the verbal report with an exhaustive list of featural descriptors may not benefit eyewitness memory. In this instance, our data suggest that more is not better. On the contrary, additional featural descriptors may inflate confidence when they appear in a description-matched lineup. However, limiting the description to only those distinctive features (should they also be replicated across all lineup members^46,47^ may reduce the diagnostic value of the lineup decision (e.g., DFD^28^.

Instead, although trait descriptors may provide little value to lineup construction due to their subjective nature, they may impart value to eyewitnesses’ memory and ability to discriminate between the perpetrator and other similar-looking individuals. Although best practices might not make room for a long list of features, additional trait descriptors may increase accuracy. Regardless of whether the eyewitness’s subjective impressions are shared by the lineup constructor (e.g., beauty is in the eye of the beholder), our data do not suggest any deleterious effect on recognition accuracy or the confidence-accuracy relationship. More research should focus on these important differences as embedded within contexts such as the cognitive interview^48^ and other conditions that guide person descriptions and their utility in both basic and applied settings.

Future research should investigate ways to instantiate VF by incorporating both types of descriptors (provided as either serial verbal reports by descriptor or in a single mixed-descriptor verbal report). Such a paradigm may create the best of both worlds, such that features are what police need but trait description boosts accuracy. Alternatively, might the disadvantages of featural description (i.e., over-confidence) overpower the advantages of trait description? These research questions are yet to be answered.

### Translating basic findings to applied settings

How might those future studies be designed to strike the important balance between internal validity (e.g., describing still images on a computer screen for a controlled amount of time in a lab room) and external validity (e.g., describing actual perpetrators under conditions that are more common in the field)? In many respects, our paradigm combines several essential aspects of a lineup (e.g., multiple, description-matched individuals at both study and test) but also adds elements that do not readily translate to the eyewitness experience. Namely, lab participants routinely describe multiple faces (in this case, 12) that are well-beyond the task demands placed on eyewitnesses. However, that is not to say that eyewitnesses cannot and perhaps should not describe multiple people. If the multiple-face paradigm more often produces VF and a single-face paradigm opens up the unwelcomed chance of verbal overshadowing (e.g.,^49^), future research should work towards uncovering how multiple faces shape the description, the study process, or both in a way that would benefit an eyewitness. Likewise, sequentially-viewed static images were essential to test the predictions of this current study. However, future studies could increase realism and test the boundary conditions of this VF effect by incorporating more realistic study (e.g., mock crime or facial videos with motion, expression changes, and environmental cues) and test (e.g., lineups with varying degrees of bias) materials. Then, researchers could help practitioners craft best practices in collecting verbal reports.

## Supplementary Information

Below is the link to the electronic supplementary material.

Supplementary Material 1

## Data Availability

The materials, data, and scripts for the current study are available in the OSF at https://osf.io/x5wue/. Data collection occurred between 2012 and 2013, and we recently returned to the project. At the time of the implementation of the study we did not pre-register as this was a newer practice, but since this time we have implemented preregistration for other studies. The data does not include time-sensitive subjects (e.g., Covid, memory for recent events); therefore, the basic memory principles observed would be consistent over time.
