# Supplementary material for: Different types of facial description alter the confidence–accuracy relationship
Source: Sci Rep. 2026 May 5;16:20668. doi: 10.1038/s41598-026-49407-0 (PMC13333856; doi:10.1038/s41598-026-49407-0)
Supplement: Supplementary file 1 — Supplementary Material 1 [file 41598_2026_49407_MOESM1_ESM.docx]

**Confidence**

A 3 (Block Order – between: Control-Control, Control-Description, or Description-Control) x 2 (Test Type – within: Inclusion and Exclusion) mixed methods factorial ANOVA on average confidence revealed a main effect of Block Order, *F*(2, 175) = 6.32, *p* = .002, *η^2^_p_* = .067, and no main effect of Test Type *F*(1, 175) = 5.54, *p* = .02 , *η^2^_p_* = .031. Importantly, there was no interaction between Block Order and Test Type, *F*(2, 175) = 2.22, *p* = .11, *η^2^_p_* = .025.

Pairwise comparisons for Block Order revealed that average confidence for Control-Control (*M* = 3.54, *SEM* = .13) was significantly lower than Description-Control (*M* = 4.1, *SEM* = .09). However, neither condition differed from Control-Description (*M* = 3.88, *SEM* = .09). In line with the main effect of Test Type, the inclusion test was relatively easier (perhaps due to stronger memories for described faces) for the two description groups.

**Confidence-Accuracy Relationship**

*Over-Under Confidence.* A 3 (Block Order – between: Control-Control, Control-Description, or Description-Control) x 2 (Test Type – within: Inclusion and Exclusion) mixed methods factorial ANOVA on average confidence revealed no main effect of Block Order, *F*(2, 175) = .38, *p* = .69, *η^2^_p_* = .004, and a main effect of Test Type *F*(1, 175) = 6.76, *p =* .01 , *η^2^_p_* = .037. Importantly, there was no interaction between Block Order and Test Type, *F*(2, 175) = .45, *p* = .64, *η^2^_p_* = .005. Lastly, pairwise comparisons for Test Type revealed that over-confidence was more pronounced for Inclusion (*M* = .04, *SEM* = .01) than Exclusion (*M* = .001, *SEM* = .01).

**Source Accuracy**

A 3 (Block Order – between: Control-Control, Control-Description, or Description-Control) x 2 (Test Type – within: Inclusion and Exclusion) mixed methods factorial ANOVA on source accuracy revealed a main effect of Block Order, *F*(2, 175) = 6.14, *p* = .003, *η^2^_p_* = .066, and a main effect of Test Type *F*(1, 175) = 8.77, *p* = .003 , *η^2^_p_* = .048. Importantly, there was no interaction between Block Order and Test Type, *F*(2, 175) = 1.30, *p* = .27, *η^2^_p_* = .015.

Pairwise comparisons for Block order revealed that source accuracy for Control-Control (*M* = .43, *SEM* = .01) was significantly lower than both Description-Control (*M* = .48, *SEM* = .01) and Control-Description (*M* = .48, *SEM* = .01), which did not differ from each other. In line with the main effect of Test Type, source accuracy for Inclusion (*M* = .48, *SEM* = .01) was significantly higher than Exclusion (*M* = .45, *SEM* = .01).
